# Case Report: Severe IgE-mediated hypersensitivity to carboxymethylcellulose with tolerance to crosscarmellose and microcrystalline cellulose

**DOI:** 10.3389/falgy.2025.1663395

**Published:** 2025-10-24

**Authors:** Rhea Schreiber, Carole Guillet

**Affiliations:** 1 Allergy Unit, Department of Dermatology, University Hospital Zurich, Zurich, Switzerland; 2 Faculty of Medicine, University of Zurich, Zurich, Switzerland

**Keywords:** carboxymethylcellulose, excipient allergy, intra-articular injection, cellulose derivatives, anaphylactic allergic reaction

## Abstract

We present a rare case of severe IgE-mediated hypersensitivity to carboxymethylcellulose (CMC) following an intraarticular knee injection with triamcinolone acetonide (Triamcort®). The patient experienced a grade IV anaphylactic reaction shortly after administration. Diagnostic workup, including skin prick testing and basophil activation test, confirmed sensitization to CMC. Importantly, the patient tolerated medications containing crosscarmellose and microcrystalline cellulose without adverse reactions, suggesting no clinically relevant cross-reactivity. This case highlights the need to consider excipients such as CMC as potential triggers of severe allergic reactions especially in cases of unexplained anaphylaxis to injectable medication and underscores the importance of thorough allergological assessment to ensure safe future treatments.

## Introduction

Carboxymethylcellulose (CMC) is a widely used pharmaceutical excipient and thickening agent in various medications, including injectable corticosteroids such as triamcinolone acetonide (*Triamcort®*). Excipient allergies are increasingly recognized as causes of severe drug hypersensitivity, yet remain underdiagnosed. Hypersensitivity reactions to CMC are rare but can be severe and life-threatening (1, 2). Reports in the literature of IgE-mediated allergy to CMC are sparse, and cross-reactivity with related cellulose derivatives such as crosscarmellose or microcrystalline cellulose remains unclear. We report a unique case of severe anaphylaxis to CMC with confirmed sensitization, while tolerance to crosscarmellose and microcrystalline cellulose was documented. This case adds important clinical insights for allergists and prescribing physicians regarding excipient allergy and cross-reactivity and this case is—to our knowledge—among the few well-documented IgE-mediated CMC allergy cases with documented tolerance to related excipients.

## Case description

A 82-year old female patient received an intraarticular knee injection for osteoarthritis with *Triamcort®* (triamcinolone acetonide), which contains carboxymethylcellulose as an excipient. The injection also included ropivacaine and hyaluronic acid (*Ostenil®*). The patient was treated for arterial hypertension with ramipril and for atrial fibrillation with apixaban (*Eliquis®)*. She had no prior anaphylactic reaction nor any history of reaction to excipients. Within minutes after intraarticular infiltration, the patient developed a severe anaphylactic reaction with generalized erythroderma, presyncope, and hypotension, requiring repeated intramuscular and intravenous adrenaline, antihistamines, corticosteroids, fluid resuscitation, and transfer to intermediate care for vasopressor support. The patient had no prior history of severe allergic reactions or known drug allergies.

Diagnostic evaluation was subsequently performed in the outpatient clinic six months after the initial reaction. It encompassed skin prick testing (SPT) with the following substances at undiluted concentrations: triamcinolone acetonide (10 mg/ml), methylprednisolone (40 mg/ml), lidocaine HCl (10 mg/ml), hyaluronic acid (*Ostenil®, 20 mg/2 ml*), carboxymethylcellulose (7.5 mg/ml) and benzyl alcohol. The carboxymethylcellulose (CMC) used for diagnostic purposes was prepared in-house by our hospital pharmacy at a concentration of 7.5 mg/mL. It is of pharmaceutical grade.

**Figure 1:**
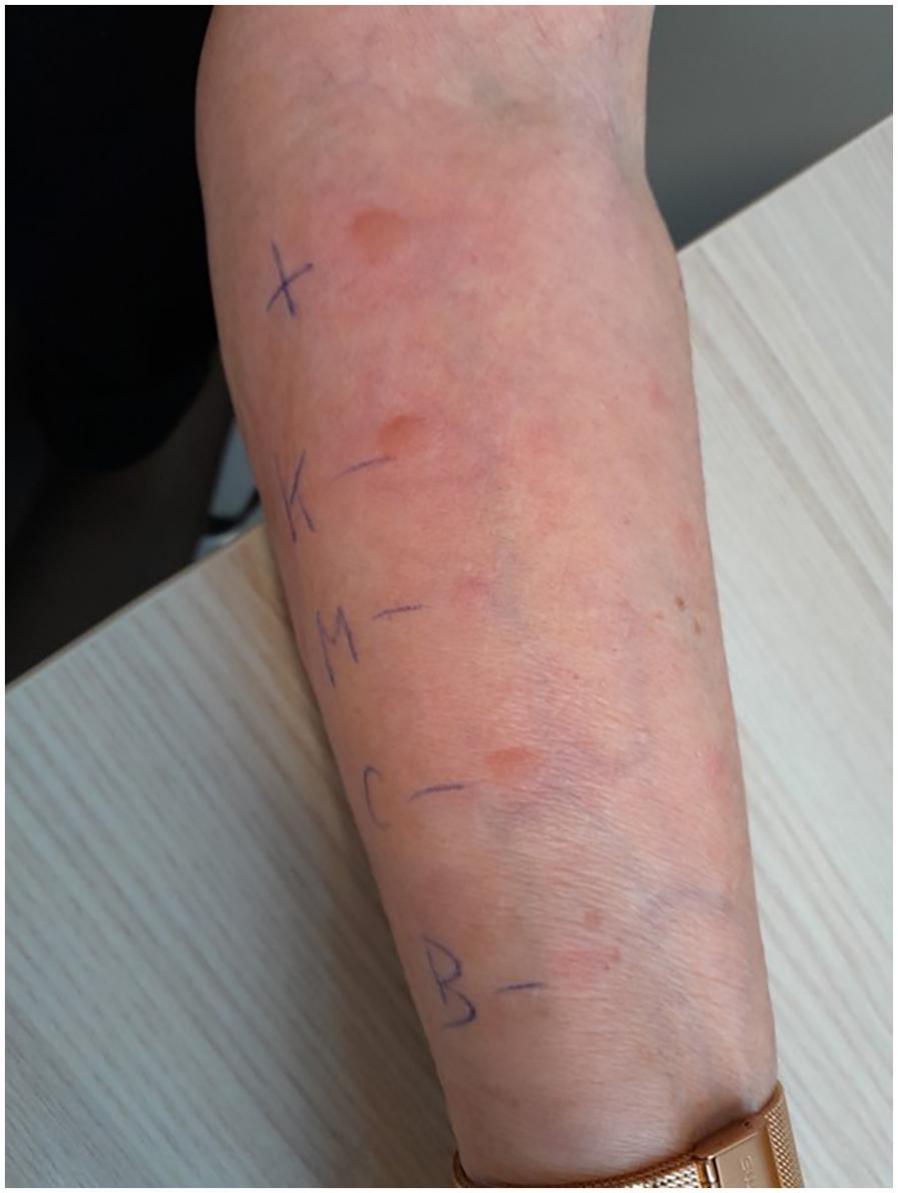
Skin prick test results demonstrating immediate-type hypersensitivity reactions. The positive control with histamine (top) elicited a strong wheal-and-flare response (++). The patient showed positive reactions to *Kenacort®* (K, ++), and to carboxymethylcellulose itself (CMC, ++) (both max. diameter of 6 mm). Test results were negative for methylprednisolone (M, –) and benzyl alcohol (BA, –).

SPT results were negative for lidocaine, triamcinolone acetonide, and methylprednisolone when tested as pure substances*. Kenacort®*, a substitute product for the originally administered *Triamcort®*, elicited a clearly positive reaction in the skin prick test, as did carboxymethylcellulose (Figure 1).

Intradermal testing was only performed with methylprednisolone at 1:100 and 1:10 dilutions, all of which yielded negative results. Intradermal testing with carboxymethylcellulose and *Kenacort®* was not performed due to the clearly positive SPT result.

**Figure 2:**
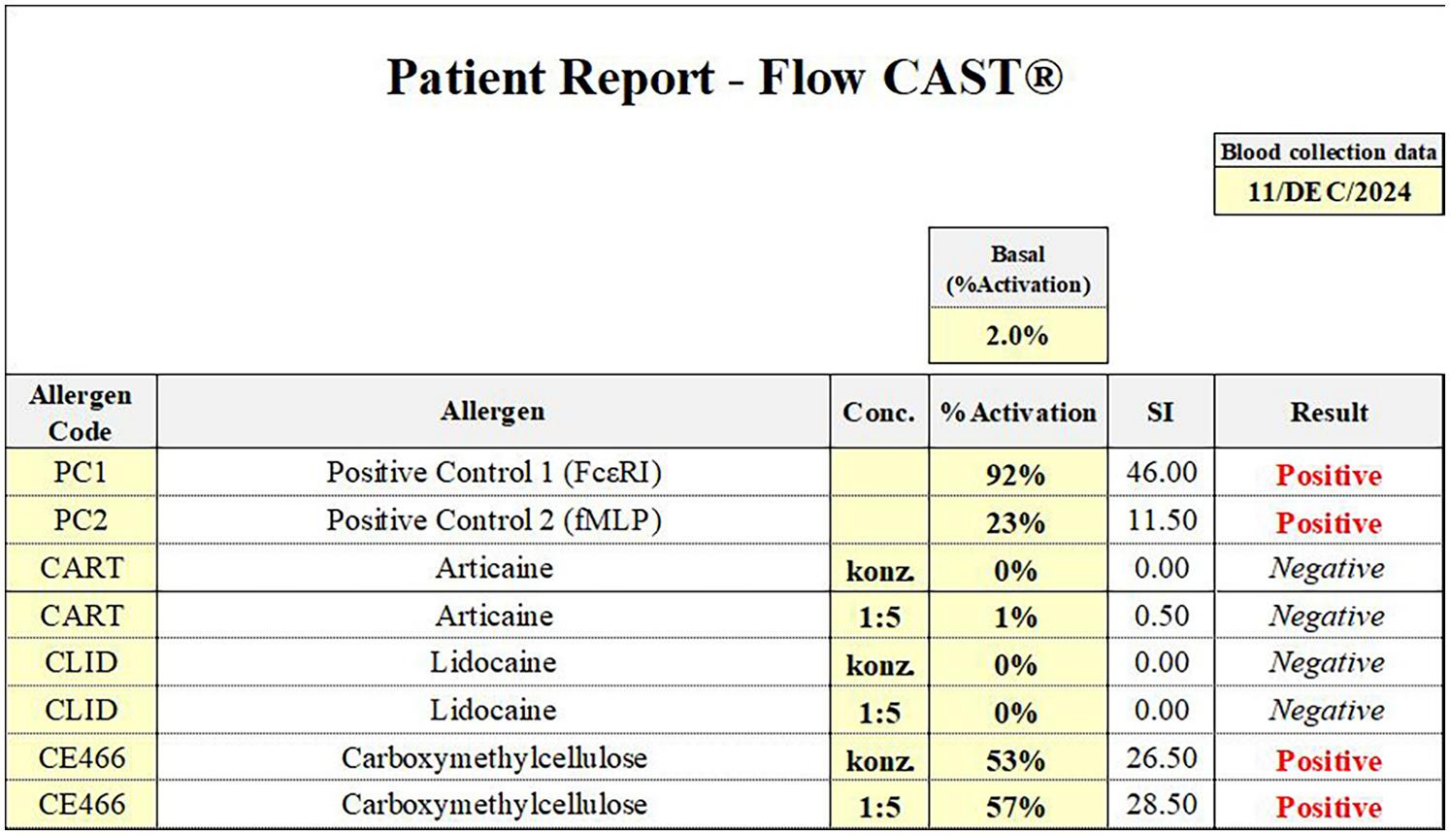
Basophil activation test (flow CAST®) results from 11 December 2024. A clearly positive response was observed to carboxymethylcellulose, while test results for lidocaine and articaine were negative. These findings support a specific hypersensitivity to carboxymethylcellulose.

Skin testing was performed using 0.9% sodium chloride as a negative control and histamine as a positive control to assess skin reactivity, in line with current recommendations (3). Skin test results are summarized in Table 1. Basophil activation testing (BAT) confirmed a positive response to carboxymethylcellulose, while results for lidocaine and articaine were negative (Figure 2). For BAT testing, the CAST Allergens from BUHLMANN Laboratories AG have been used according to the manufacturer's instructions.

**Table 1:** Skin test results.

Skin testing			
Substance	Concentration	Prick testing (dilution)	Intradermal testing (dilution)
Triamcinolone acetonide	10 mg/ml	++ (1:1)	Not done
Methylprednisolone	40 mg/ml	Negative (1:1)	Negative (1:10; 1:100)
Lidocain HCl	10 mg/ml	Negative (1:1)	Negative (1:5)
Ostenil (hyaluronic acid)	20 mg 72 ml	Negative (1:1)	Not done
Benzyl alcohol	20 mg/ml	Negative (1:1)	Not done
Histamine (positive control)	10 mg/ml	++	++
NaCl 0.9% (negative control)	0.90%	–	–

Serum tryptase and total IgE were within normal limits (5.46 µg/L and 16.7 kU/L, respectively). No specific IgE to chlorhexidine or latex was detected. Additional provocation tests in May 2025 showed the patient tolerated subcutaneous administration of ropivacaine and mepivacaine as well as oral methylprednisolone (*Solu-Medrol®* 125 mg), confirming no sensitization to these alternative drugs. These oral challenges were performed on a specialized allergy ward under strict medical supervision, where continuous monitoring and immediate resuscitation measures were available in case of an adverse reaction, in accordance with established safety protocols and ethical standards for drug provocation testing in patients with a history of severe hypersensitivity reactions.

Given the suspected excipient allergy, medications containing carboxymethylcellulose (also known as Carmellose or E466) were strictly avoided. Interestingly, the patient tolerated *Eliquis®* tablets (apixaban) containing microcrystalline cellulose and crosscarmellose well, indicating no cross-reactivity to these related cellulose derivatives. This clinical observation suggests that despite structural similarity, these excipients may differ immunologically, allowing safe use in this patient.

Diagnostic tests confirmed IgE-mediated hypersensitivity to carboxymethylcellulose. Skin prick testing and BAT were essential to establish the diagnosis. Negative tests for local anesthetics and corticosteroids allowed safe administration of these agents, reducing future anaphylaxis risk. The patient was provided with a detailed allergy passport specifying avoidance of carboxymethylcellulose-containing drugs.

The reaction occurred in February 2024, and the evaluation was completed in July 2025. During this follow-up period, the patient experienced no further reactions upon subsequent exposures.

## Discussion

This case illustrates a rare but critical hypersensitivity to carboxymethylcellulose as an excipient causing severe anaphylaxis. Few cases have been described, and awareness of excipient allergies remains low in clinical practice. Our findings emphasize the importance of including excipients in the allergy workup, especially when reactions occur after administration of well-known drugs.

When unexplained hypersensitivity occurs after administration of multi-component medications, excipient allergy should be considered, and testing for specific excipients such as CMC should be performed.

The lack of clinical reactivity to crosscarmellose and microcrystalline cellulose in our patient aligns with current evidence suggesting that these cellulose derivatives do not exhibit clinically relevant cross-reactivity with carboxymethylcellulose (CMC) in the context of IgE-mediated allergy.

Although both CMC and croscarmellose sodium are chemically related cellulose derivatives, they differ significantly in structure and physicochemical properties: CMC is a linear, water-soluble anionic polymer, whereas croscarmellose is a cross-linked, water-insoluble derivative used primarily as a disintegrant in oral solid formulations (4). These structural differences between CMC and other cellulose derivatives may explain the observed lack of cross-reactivity. The European Food Safety Authority (EFSA) highlights that, although a “read-across” between different cellulose derivatives is possible due to structural and biological similarities there is no evidence of specific cross-reactions (5).

Previous reports have demonstrated that patients with confirmed IgE-mediated anaphylaxis to carboxymethylcellulose, triggered by triamcinolone acetonide injections, tolerated oral administration of trimethoprim-sulfamethoxazole (which contains small amounts of CMC) as well as oral provocation with typical dietary and pharmaceutical doses of CMC, suggesting an absence of clinically relevant reactivity via the oral route (1, 6, 7). Due to the severity of the initial reaction, we refrained from performing an oral provocation test with CMC in our patient.

Limitations of this report include its single-patient nature, which precludes generalizability. Moreover, we did not perform a direct oral provocation test with carboxymethylcellulose due to the severity of the initial anaphylactic reaction (grade IV) and the associated ethical and safety considerations. While this limits the ability to definitively exclude oral reactivity, the clear positive results of skin testing and basophil activation testing, together with the absence of any reaction to structurally related cellulose derivatives (crosscarmellose and microcrystalline cellulose) during routine treatment, provide strong indirect evidence supporting selective sensitization to CMC.

Although the patient tolerated croscarmellose sodium and microcrystalline cellulose in Eliquis®, the amounts of these excipients per tablet are very low and likely below the threshold required to elicit a clinical reaction. However, repeated low-dose exposures could, in theory, contribute to ongoing sensitization or subclinical immune activation, a phenomenon that has been reported in other excipient allergies.

Another limitation is the lack of *in vitro* IgE quantification specific to CMC, as such assays are not commercially available. While the diagnostic workup included both skin testing and functional cellular assays, the immunological mechanism—although highly suggestive of IgE-mediated allergy—cannot be fully confirmed at a molecular level. To address the inherent limitation of this single-case report, future research should build on existing protocols or patient registries dedicated to rare drug hypersensitivities, providing a framework for multicenter studies and the aggregation of data from larger patient cohorts. Such collaborative efforts would help to validate and extend the findings observed in this patient and improve understanding of excipient-induced hypersensitivity.

## Data Availability

The original contributions presented in the study are included in the article/Supplementary Material, further inquiries can be directed to the corresponding author.
